# Supplementary material for: Expertise based skills management system to support resource allocation
Source: PLoS One. 2021 Aug 16;16(8):e0255928. doi: 10.1371/journal.pone.0255928 (PMC8367002; doi:10.1371/journal.pone.0255928)
Supplement: S1 Appendix — Results of the survey, Questioner used for the evaluation of developed tool. Finally, the results of evaluation survey are also enclosed in the file. (PDF) [file pone.0255928.s001.pdf]

S1 Appendix

A. Data Collection Questioner

The questionnaire as shown in Figure S1-A and S1-B is used to gather data about the skills management and resource allocation relation with skill management practices. The questionnaire consists of five parts. There are 27 open ended and closed questions. Each question has a specific purpose. The purpose of each section of this questionnaire is explained in paper. This questionnaire is designed after extensive literature review and the brainstorming sessions. We performed context analysis on literature and extracted different terms that are related to skills management, expertise, skills assessment, skills rating, performance management, resource allocation and practices for resource allocation. After this, people from different areas are invited in brainstorming sessions to formulate different questions and these questions are grouped in five sections.

Questionnaire for Skill Management & Resource Allocation Relation Assessment

Job Role: ________________ **Experience:** __________ **Highest Qualification:** ______________

Skills Assessment Questions (For verification of concept)

Questions	Strongly Agree	Agree	Neutral	Disagree	Strongly Disagree
Your employees possess multiple skills in addition to their job role?	☐	☐	☐	☐	☐
Skills must be rated based on expertise of employees?	☐	☐	☐	☐	☐
You use additional skills of employees working under you?	☐	☐	☐	☐	☐
You think that Emotional Intelligence can be used to judge expertise of employees?	☐	☐	☐	☐	☐

Your existing practice for Skills Management?			
Using Job Description	☐	Skill Management System	☐
Expertise Based Skill Management	☐	Any Other : ____________	☐

Skills Rating Questions (To propose a system for skills rating)

**What should be scale for skills rating?**			
On a scale of High, Medium and Low?	☐	On a scale of 1 to 10 (1 for low and 10 for high)	☐
On a scale of Expert, Intermediate and Beginner?	☐	Any Other : ____________	☐
**How do you measure skills of employees working under you?**			
Through Knowledge	☐	Through Experience	☐
Previous and Current Projects	☐	Any Other : ____________	☐
**In your opinion how to rate / grade skills of human resources?**			
Performance Evaluation	☐	Quality of Work	☐
Success Rate	☐	Any Other : ____________	☐

Resource Allocation Questions (To understand existing method of resource allocation)

**How do you allocate human resources to projects?**			
Job Description	☐	Expertise	☐
Success Rate	☐	Any Other : ____________	☐

**What problems are you facing while allocating human resources to projects?**			
Finding appropriate employees	☐	Lack of skills record	☐
Not enough resources to allocate	☐	Other : ____________	☐
**Do you assign employees to unrelated jobs in case of resource conflict?**			

Fig S1-A. Questionnaire-Page 1.

Skills based Resource Allocation (To verify that skills data is useful for resource allocation)

Questions	Yes	NO
You use any software tool for skills management?	☐	☐
You need any skills management software?	☐	☐
You think that skill management software will contribute to proper resource utilization?	☐	☐
Skill management tool/software, increase organization performance and efficiency?	☐	☐
Proper skill management helps in project management?	☐	☐
For effective project resource utilization a competency based skill model is necessary?	☐	☐
Skill management system must be integrated into project management software?	☐	☐

Skill Management Model Requirements (Please provide your specific Requirements)

Requirements / Features	Strongly Agree	Agree	Neutral	Disagree	Strongly Disagree
Module for Educational Record of Employee	☐	☐	☐	☐	☐
Module to enter Work Experience of Employee	☐	☐	☐	☐	☐
Based on Skills Rating	☐	☐	☐	☐	☐
Expertise Based Ranking	☐	☐	☐	☐	☐
Success record of Current and Previous Projects	☐	☐	☐	☐	☐
Employee Training Record	☐	☐	☐	☐	☐
Employee Job Description Record	☐	☐	☐	☐	☐
Input from Emotional Intelligence of Manager	☐	☐	☐	☐	☐
Integration with Project Management Software for Project Resource Allocation	☐	☐	☐	☐	☐
Other: ____________________	☐	☐	☐	☐	☐
Other: ____________________	☐	☐	☐	☐	☐
Other: ____________________	☐	☐	☐	☐	☐
Other: ____________________	☐	☐	☐	☐	☐

Where skills data must be shown in project management software?

In Resource Calendar	☐	In Resource Sheet	☐
As separate view in Project Management Software	☐	Other :____________________	☐

Your Comments:

Fig S1-B. Questionnaire-Page 2.

B. Results of Survey

In In this appendix, we show the results of analysis that is performed on the response of questionnaire in the form of graphs. On the basis of these results, we propose and develop the skills calculating framework and the supporting tool. An overview of the results is already given in paper.

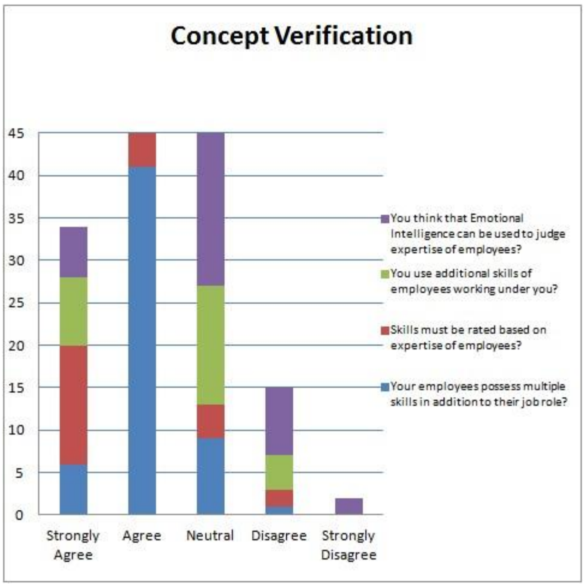

(a) Concept Verification

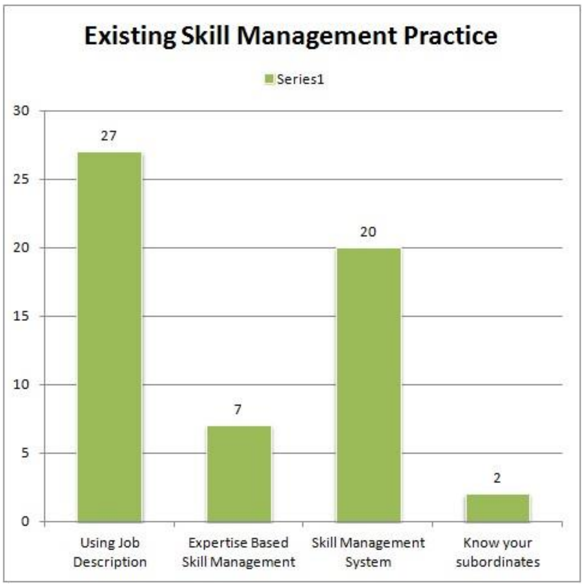

(b) Skill Management Practice

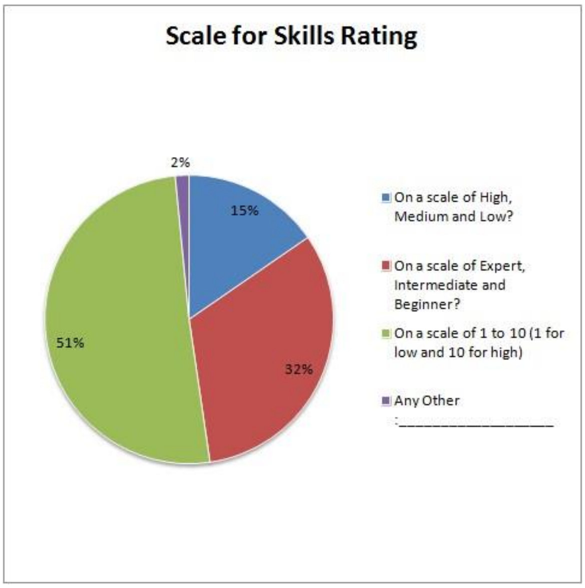

(c) Scale for Skill Rating

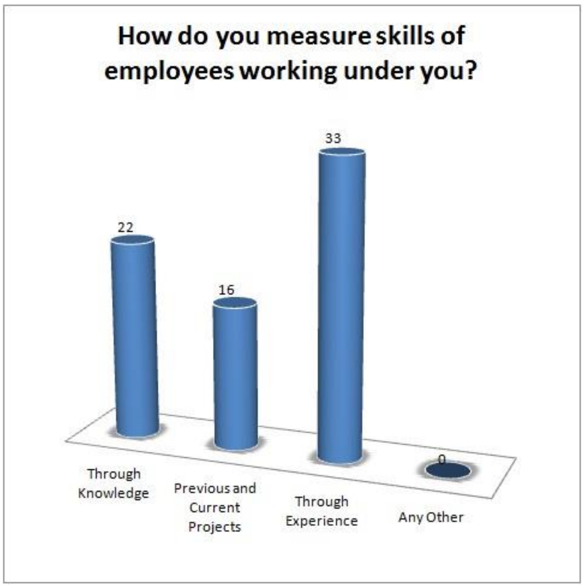

(d) Current Practice for Skill

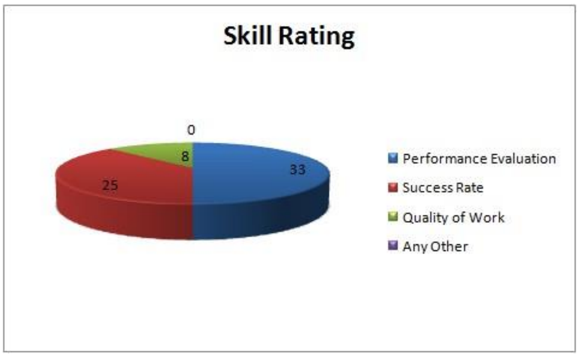

(e) Skill Rating

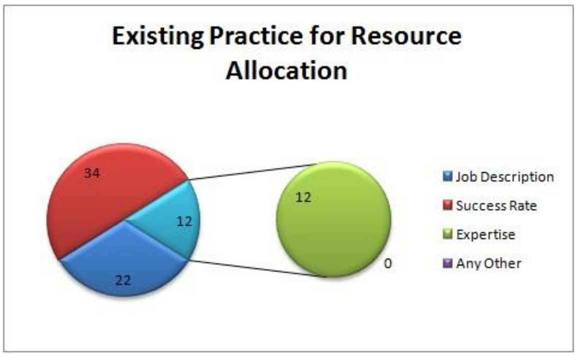

(f) Resource Allocation Practice

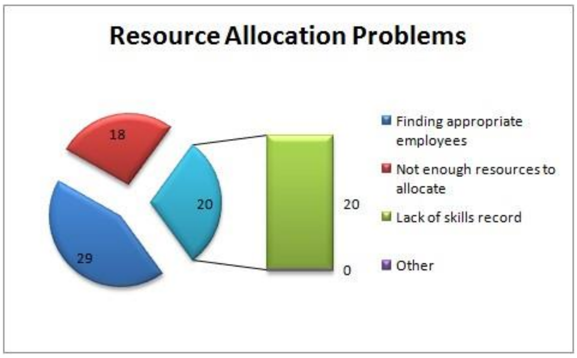

(g) Problems in Resource Allocation

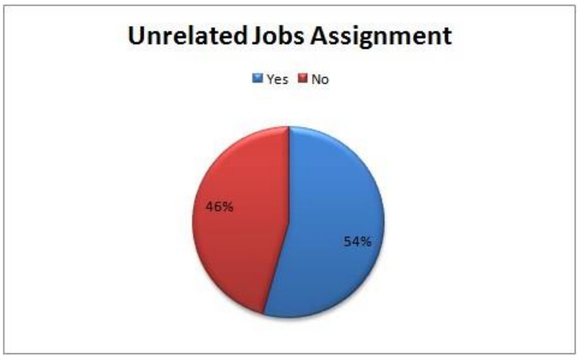

(h) Un-Related Job Assignment

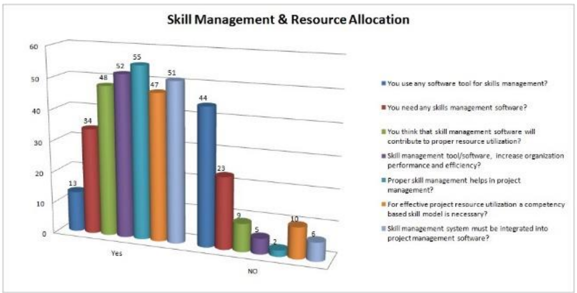

(i) Skills Management and Resource Allocation

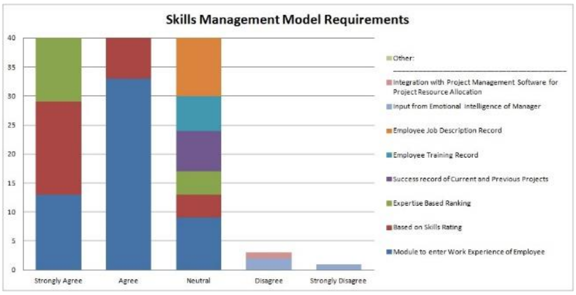

(j) Skills Management Model Requirements

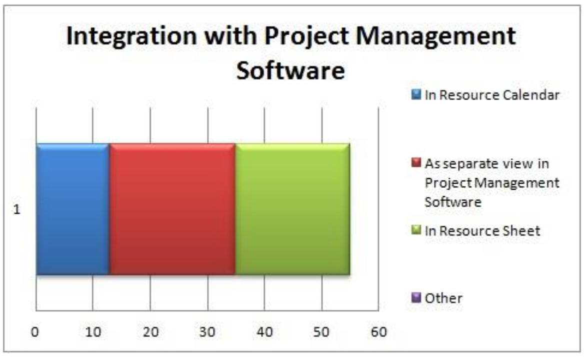

(k) Integration with Project Management Software

C. Evaluation Questioner

Questionnaire that we used for the evaluation of our developed framework and tool is given in Figure S3-A and Figure S3-B. We used three software evaluation surveys that are available online to design this questionnaire. The web links of these surveys are given below:

https://www.surveymonkey.com/mp/software-evaluation-survey-template/
https://www.survio.com/survey-template/software-evaluation-survey
https://www.questionpro.com/survey-templates/software-evaluation/

Software Evaluation

EXPERTISE BASED SKILL MANAGEMENT SYSTEMS (EBSMS)

Name: ____________________

Designation: ____________________ **Experience:** ____________________

1. What is your overall feeling about the performance of (Expertise Based Skill Management Software)?

☐ Very Satisfied
☐ Somewhat Satisfied
☐ Neutral
☐ Somewhat Unsatisfied
☐ Very Unsatisfied

2. **Usefulness** of "Expertise Based Skill Management" Software

	Strongly Agree	Agree	Neutral	Disagree	Strongly Disagree
It helps me be more effective.	☐	☐	☐	☐	☐
It helps me be more productive and improves my job performance	☐	☐	☐	☐	☐
It is useful in my job	☐	☐	☐	☐	☐
It makes the things I want to accomplish easier to get done.	☐	☐	☐	☐	☐
It saves my time when I use it.	☐	☐	☐	☐	☐
It meets my needs.	☐	☐	☐	☐	☐
It makes it easier to do my job.	☐	☐	☐	☐	☐

3. **Ease of Use** when using "Expertise Based Skill Management" software

	Strongly Agree	Agree	Neutral	Disagree	Strongly Disagree
It is easy to use.	☐	☐	☐	☐	☐
It is user friendly.	☐	☐	☐	☐	☐
It requires the fewest steps possible to accomplish what I want to do with it.	☐	☐	☐	☐	☐
I can use it without written instructions.	☐	☐	☐	☐	☐
I don't notice any inconsistencies as I use it.	☐	☐	☐	☐	☐
I can recover from mistakes quickly and easily.	☐	☐	☐	☐	☐
I can use it successfully every time.	☐	☐	☐	☐	☐
My interaction with EBSMS would be clear and understandable.	☐	☐	☐	☐	☐
EBSMS is flexible to interact with.	☐	☐	☐	☐	☐

4. **Ease of Learning** "Expertise Based Skill Management" Software

	Strongly Agree	Agree	Neutral	Disagree	Strongly Disagree
I learned to use it quickly.	☐	☐	☐	☐	☐
I easily remember how to use it.	☐	☐	☐	☐	☐
I quickly became skillful with it.	☐	☐	☐	☐	☐

5. Employees **Satisfaction** to work with "Expertise Based Skill Management" Software

	Strongly Agree	Agree	Neutral	Disagree	Strongly Disagree
It works the way I want it to work.	☐	☐	☐	☐	☐
I feel, I need to have it.	☐	☐	☐	☐	☐

6. Answer the following in the light of **System Usability** :

	Strongly Agree	Agree	Neutral	Disagree	Strongly Disagree
I think that I would like to use EBSM frequently	☐	☐	☐	☐	☐
I found the system unnecessarily complex	☐	☐	☐	☐	☐
I think that I would need the support of a technical person to be able to use this system	☐	☐	☐	☐	☐
I found that various functions in this system were well integrated	☐	☐	☐	☐	☐
I thought there was too much inconsistency in this system	☐	☐	☐	☐	☐
I would imagine that most people would learn to use this system very quickly	☐	☐	☐	☐	☐
I found the system very cumbersome to use	☐	☐	☐	☐	☐
I feel confident using the system	☐	☐	☐	☐	☐
I needed to learn a lot of things before using this system	☐	☐	☐	☐	☐

Fig S3-C Questionnaire-Page 1.

7. **Performance expectancy** of software

	Strongly Agree	Agree	Neutral	Disagree	Strongly Disagree
I would find the system useful in my job.	☐	☐	☐	☐	☐
Using the system enables me to accomplish tasks more quickly.	☐	☐	☐	☐	☐
Using the system increases my productivity.	☐	☐	☐	☐	☐

8. **Effort expectancy** of software

	Strongly Agree	Agree	Neutral	Disagree	Strongly Disagree
My interaction with the system is clear and understandable.	☐	☐	☐	☐	☐
It would be easy for me to become skillful at using the system.	☐	☐	☐	☐	☐
I find the system easy to use.	☐	☐	☐	☐	☐
Learning to operate the system is easy.	☐	☐	☐	☐	☐

9. **Attitude toward using technology (or using this software)**

	Strongly Agree	Agree	Neutral	Disagree	Strongly Disagree
Using the system is a good idea.	☐	☐	☐	☐	☐
The system makes work more interesting.	☐	☐	☐	☐	☐
I feel excited to work with the system.	☐	☐	☐	☐	☐
I like working with the system.	☐	☐	☐	☐	☐

10. **Self-efficacy**
I could complete a job or task using the system...

	Strongly Agree	Agree	Neutral	Disagree	Strongly Disagree
If there was no one around to tell me what to do as I go.	☐	☐	☐	☐	☐
If I could call someone for help if I got stuck.	☐	☐	☐	☐	☐
If I had a lot of time to complete the job for which the software was provided.	☐	☐	☐	☐	☐
If I had just the built-in help facility for assistance.	☐	☐	☐	☐	☐

11. **Please rate your level of satisfaction with the following aspects of our software:**

	Very Satisfied	Somewhat Satisfied	Neutral	Somewhat Unsatisfied	Very Unsatisfied
Ease of Installation	☐	☐	☐	☐	☐
Hardware Compatibility	☐	☐	☐	☐	☐
Operating System Compatibility	☐	☐	☐	☐	☐
Consistency with Interface	☐	☐	☐	☐	☐
Clarity of Documentation	☐	☐	☐	☐	☐
Overall Reliability	☐	☐	☐	☐	☐
Overall Performance	☐	☐	☐	☐	☐
Please add any additional comments/suggestions:					

12. How likely are you to recommend our software to others?

☐ Highly likely 100%
☐ Quite likely 75%
☐ Maybe yes
☐ Maybe no
☐ Not very likely
☐ Wouldn't recommend it to anyone

Fig S1-D. Questionnaire-Page 2.

S4. Results of Evaluation Survey

In this appendix, we show the results of analysis in the forms of graphs. These graphs are made as a result of the responses received from the survey respondents. On the basis of these results, we evaluate the developed framework and tool.

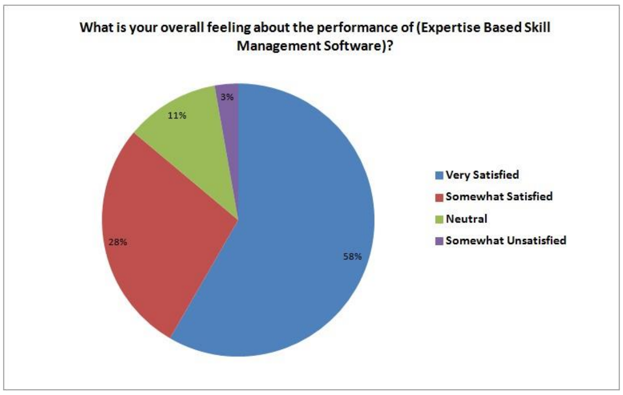

(a) Performance of EBSM

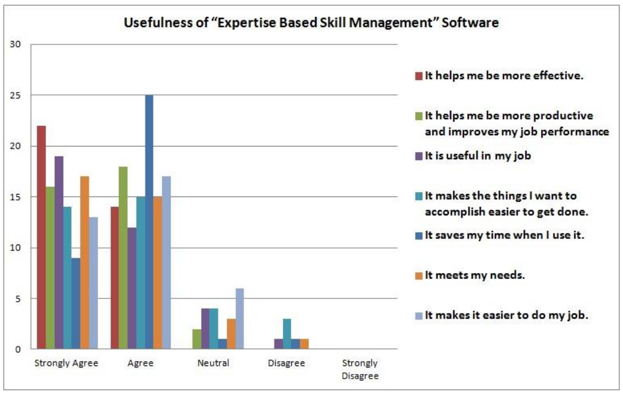

(b) Usefulness of EBSM

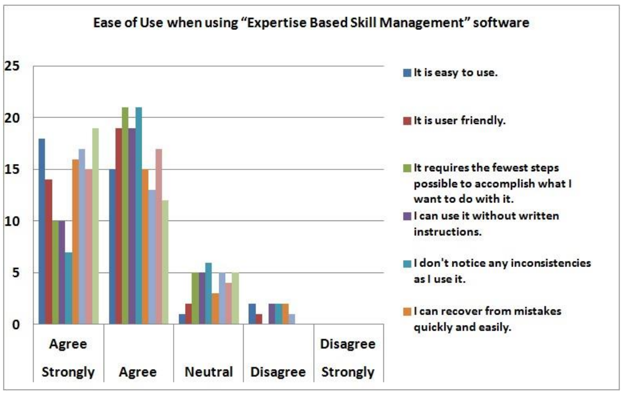

(c) Ease of Use

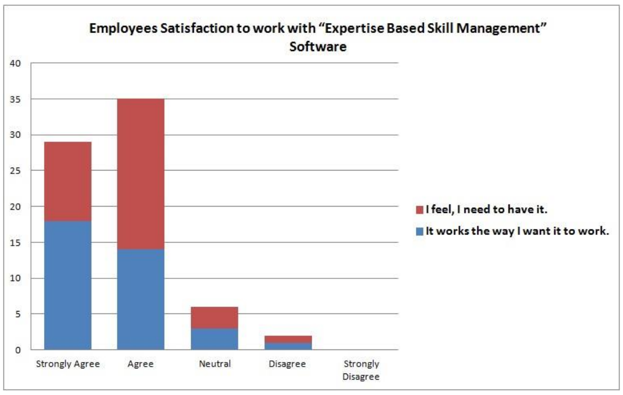

(d) Employee Satisfaction

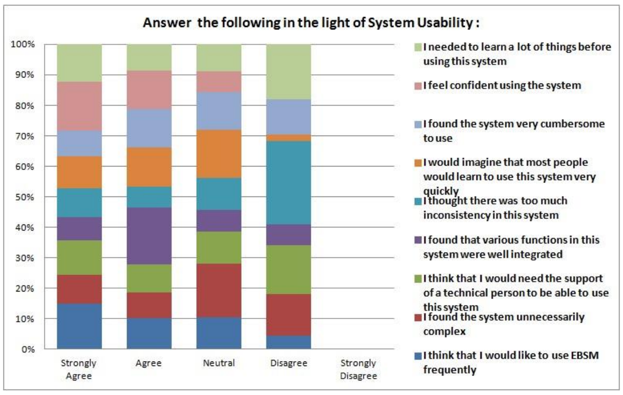

(e) System Usability

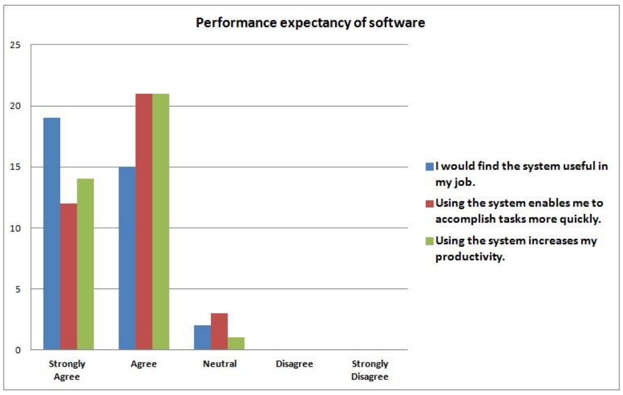

(f) Performance

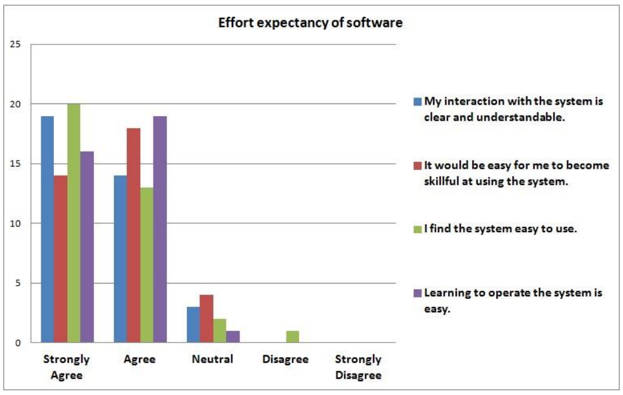

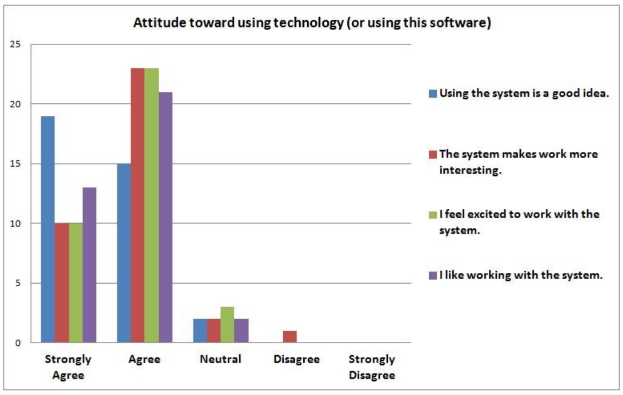

(g) Usefulness of EBSM

(h) Ease of Use

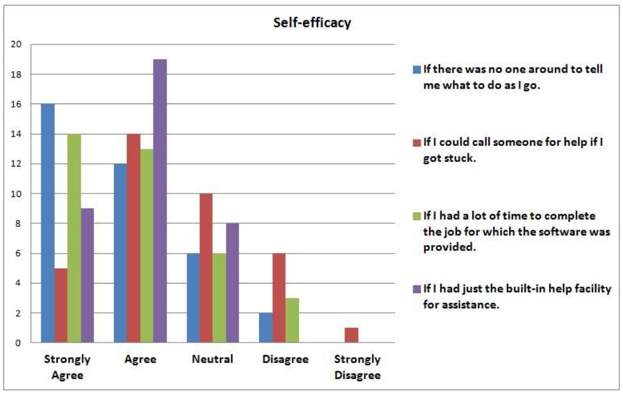

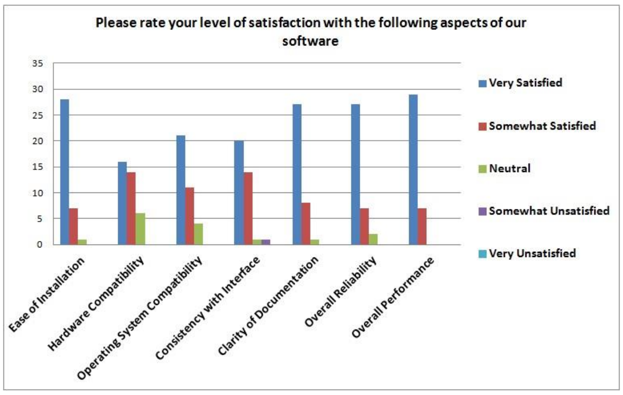

(i) Employee Satisfaction

(j) System Usability

a
