# Supplementary material for: Expertise based skills management system to support resource allocation
Source: PLoS One. 2021 Aug 16;16(8):e0255928. doi: 10.1371/journal.pone.0255928 (PMC8367002; doi:10.1371/journal.pone.0255928)
Supplement: Attachment — Submitted filename: ethics statement.pdf [file pone.0255928.s002.pdf]

PLOS ONE

Manuscript Title: Expertise based Skills Management System to Support Resource Allocation

Manuscript ID: PONE-D-20-22779

Authors: Nazia Bibi, Zeeshan Anwar and Tauseef Rana

Ethics statement

On behalf of the authors of this paper and having obtained permission from all the authors, I declare that during collecting data from various participants in the software industry of Pakistan, this study completely followed the user agreement and privacy of the users and organization. The collection method also complied with the terms and conditions for the organizations and university. Besides, all the personal and organizational information of participants involved in the cases is protected and not reported in the paper.

I testify to the accuracy of the above on behalf of all the authors

Zeeshan Anwar

August 05, 2020
