# Supplementary material for: Expertise based skills management system to support resource allocation
Source: PLoS One. 2021 Aug 16;16(8):e0255928. doi: 10.1371/journal.pone.0255928 (PMC8367002; doi:10.1371/journal.pone.0255928)
Supplement: Attachment — Submitted filename: Response to Reviewers.doc [file pone.0255928.s003.doc]

**Paper Title:** Expertise based Skills Management System to Support Resource Allocation

**Paper ID:** PONE-D-20-22779

Dear Sir,

We are very thankful to you for performing an in-depth review of the manuscript and raising the important issues. The raised issues were very logical and their incorporation was essentially needed to transform a manuscript into a comprehensive study. These proposed changes really helped us to improve the quality of the paper. We have carefully understood the comments and have substantially revised our paper to address your concerns. Changes in the paper are highlighted as red color. The following table explains the incorporation status, response to each comment and reference to a part of manuscript where the suggestions have been incorporated.

We again thank you and waiting for your response for publication of this paper in PLOS-One.

Sincerely,

Zeeshan Anwar

PhD Scholar

Department of Computer Software Engineering,

National University of Sciences and Technology (NUST),

Islamabad, 41000, Pakistan

zeeshan.phdcse@students.mcs.edu.pk

Reviewer: Neural Computing, IEEE Access, Scientometrics, JABM, IAJ, IJSE, PONE, Springer Nature

| **Sr. #** | **Comments** | **Incorporation**  **Status** | **Response** | **Page effected** |
| --- | --- | --- | --- | --- |
| **Reviewer: 1** | | | | |
| **1** | The paper has presented one of the quite important model for the skill assessment. However, it lack critical review in that area. | **Done** | We have changed the related work section and added critical review of existing literature. | 3, 4, 17 & 18 |
| **2** | The paper intends to propose a kind of a decision support tool that can help managers to perform skills assessment towards their employees. Hence, more literature on that area should be provided. | **Done** | We have added more literature and re-organized the related work section to address this concern. | 3, 4, 5 |
| **3** | The basis and the literature review provided may not be sufficient to show how the model is developed. | **Done** | We have added / highlighted the literature and features that are proposed in the literature which help us to develop the model. | 5 |
| **4** | The whole description of the proposed model and how the model is tested can be described in a technical report or a thesis. AT present, it is quite difficult to present the proposed model as a paper without having to say that there are still many missing information about the proposed model. | **Done** | This paper is outcome of a complete thesis. You are right whole thesis cannot be described in the paper. We have tried our best to add the missing information and complete steps that describe the proposed model. | - |
| **5** | It is recommended that the paper just focused on one important aspect of the proposed model and the authors are able to give the details description of that particular aspect rather than having quite a long general description about the whole model development and evaluation. | **Done** | The main focus of this paper is to describe the framework proposed in the paper. We have shorten the model development and evolution sections. Un-necessary details is deleted from the abstract, introduction and proposed framework sections. | 1, 2 & 7 |
| **Reviewer: 2** | | | | |
| **1** | The authors claim that there is an extreme lack of available tools to support for monitoring and evaluating human skills, and conduct a survey across various software organizations to find out the best practices for skill management and to gather requirements from software development industry.  While this is a considerable effort, I find that this study did not conduct a thorough literature review as there is significant research on competence analysis and management such as:   Bohlouli, Mahdi, et al. "Competence assessment as an expert system for human resource management: A mathematical approach." Expert Systems with Applications 70 (2017): 83-102.   Could the authors elaborate on how the presented work is different from the work presented above where social competence is also considered? And in any case, include competence management in the related work as they are related to the same topic. | **Done** | The literature review section is updated as per the raised concerns and more related papers are added in literature review along-with the critical review of existing literature.  We have gone through the suggested paper. We found it very useful and added review of proposed paper. One of the main point raised in this paper is that it uses the combination of software technologies and mathematical and statistical methods for assessing and analyzing competences in human resource information systems. This point also has depicted from our work. | 3, 4, 5 13, 14, 16, 17 & 18 |
| **2** | Minor issues: -I could not find any reference to Eq (1) in the manuscript (page 3). -Short url is not working (page 3). | **Done** | Missing reference to Eq (1) is added. We have removed the short url links and added full links. | Page 3 |
