# Supplementary material for: Expertise based skills management system to support resource allocation
Source: PLoS One. 2021 Aug 16;16(8):e0255928. doi: 10.1371/journal.pone.0255928 (PMC8367002; doi:10.1371/journal.pone.0255928)
Supplement: Attachment — Submitted filename: Response to Reviewers.doc [file pone.0255928.s004.doc]

**Paper Title:** Expertise based Skills Management System to Support Resource Allocation

**Paper ID:** PONE-D-20-22779R1

Dear Sir,

We are very thankful to you for performing an in-depth review of the manuscript and accepting the changes that we made in the revised version. The editor has proposed the changes in the paper to make it more valuable and align with the PONE format. The raised issues were very logical and their incorporation was essentially needed to transform a manuscript into a comprehensive study. These proposed changes really helped us to improve the quality of the paper. We have carefully understood the comments and have substantially revised our paper to address your concerns. Changes in the paper are highlighted as red color. The following table explains the incorporation status, response to each comment and reference to a part of manuscript where the suggestions have been incorporated.

We again thank you and waiting for your response for publication of this paper in PLOS-One.

Sincerely,

Zeeshan Anwar

PhD Scholar

Department of Computer Software Engineering,

National University of Sciences and Technology (NUST),

Islamabad, 41000, Pakistan

zeeshan.phdcse@students.mcs.edu.pk

Reviewer: Neural Computing, IEEE Access, Scientometrics, JABM, IAJ, IJSE, PONE, Springer Nature

| **Editor’s Review** | | | | |
| --- | --- | --- | --- | --- |
| **Sr. #** | **Comments** | **Incorporation**  **Status** | **Response** | **Page effected** |
| **1** | The paper still needs a thorough professional mother tongue proofreading | **Done** | The paper was proof read by an Australian PR and dozens of language mistakes are correct. The corrections are highlighted in red color in the revised paper. | All Paper |
| **2** | An accurate check with the PLOS One formatting guidelines (for structure, style and reference format). | **Done** | We sub-divided this comment into three parts and our response again each part is given below: |  |
| **2(a)** | Structure | **Done** | We followed the PONE latex template available at PONE site and also re-organized the paper to improve its appearance. | Various pages |
| **2(b)** | Style | **Done** | We downloaded few PONE recently published papers and changed the style of paper as per recently published papers. Following changes are made:  Change Figures  Update Figures captions  Align figures  Update Tables Captions  Changed table format  Removed footnotes | Various pages. |
| **2(c)** | Reference Format | **Done** | We changed / corrected the references to align with PONE style. | Reference section |
